# Examining equity-related eligibility criteria in clinical trials supporting 2022 US drug approvals

**DOI:** 10.1371/journal.pone.0324807

**Published:** 2025-06-25

**Authors:** Kathy Sliwinski, Jennifer E. Miller, Holly Fernandez Lynch

**Affiliations:** 1 Center for Health Services and Outcomes Research, Northwestern University Feinberg School of Medicine, Chicago, Illinois, United States of America; 2 Center for Health Outcomes and Policy Research, University of Pennsylvania School of Nursing, Philadelphia, Pennsylvania, United States of America; 3 Internal Medicine, Yale School of Medicine, New Haven, Connecticut, United States of America; 4 Biomedical Informatics and Data Science, Yale School of Medicine, New Haven, Connecticut, United States of America; 5 Program for Biomedical Ethics, Yale School of Medicine, New Haven, Connecticut, United States of America; 6 Department of Medical Ethics and Health Policy, Perelman School of Medicine, University of Pennsylvania, Philadelphia, Pennsylvania, United States of America; Montclair State University, UNITED STATES OF AMERICA

## Abstract

There has been substantial recent attention to the importance of diversity in clinical trials supporting US Food and Drug Administration (FDA) drug approvals, including both Congressional action and FDA guidance. One factor that can contribute to a lack of diverse enrollment is overly strict eligibility criteria not supported by scientific or safety considerations. The aim of this study was to examine equity-related eligibility criteria in pivotal trials supporting all new molecular entities and therapeutic biologics approved by FDA’s Center for Drug Evaluation and Research in 2022. We qualitatively coded these criteria, using a codebook developed through inductive and deductive methods. The codebook consisted of categories relevant to equitable inclusion across race, ethnicity, socioeconomic status, pregnancy, disability, and age; explanations of the potential relevance to equity, as well as potential scientific and regulatory justifications. We found that adolescents, pregnant/lactating individuals, and individuals with limited literacy were most commonly excluded and that about 1 trial in 5 excluded individuals based on weight/obesity, had general exclusions for hepatitis, or excluded adults aged 80 and up. Ultimately, our results indicate that several common eligibility criteria are likely to negatively influence equitable inclusion, especially based on age, pregnancy/lactation, and characteristics associated with race, ethnicity, and socioeconomic status. To minimize unnecessary equity-related exclusions, eligibility should focus on specific issues expected to influence safety or efficacy (e.g., liver function, drug interaction, uncontrolled comorbid disease), rather than blanket exclusion based on broad diagnoses or characteristics. Investigators, sponsors, institutional review boards, and regulators should scrutinize eligibility criteria with equity in mind.

## Introduction

Because clinical trials evaluate medical product safety and efficacy in support of regulatory and clinical decisions, participants should reflect target populations across clinically relevant characteristics. Diverse enrollment can also promote trust and fair distribution of benefit [1]. However, certain populations—racial, ethnic, and linguistic minorities; adolescents and older adults; pregnant people; individuals with disabilities; and individuals of low socioeconomic status—are often underrepresented in trials [2]. Because overly strict eligibility criteria not justified by scientific need or participant safety can contribute to this problem, we examined eligibility criteria that may impact equitable inclusion in trials supporting US Food and Drug Administration (FDA) drug approvals.

## Methods

We examined pivotal trials supporting all new molecular entities and therapeutic biologics approved by FDA’s Center for Drug Evaluation and Research (CDER) in 2022 [3]. We selected this year given increased attention to trial diversity, including a 2022 FDA draft guidance document and federal legislation requiring diversity action plans [4]; accordingly, 2022 approvals based on earlier pivotal trials can provide a baseline against which future approvals may be assessed. We identified pivotal trials by reviewing summary and/or medical review documents in approval packages posted to the Drugs@FDA website. We matched these trials on Clinicaltrials.gov using their National Clinical Trials (NCT) identification numbers and extracted language describing eligibility criteria.

We then qualitatively coded these criteria, using a codebook developed through inductive and deductive methods, beginning with broad, pre-defined categories (e.g., exclusion based on age), then refining and expanding codes based on the data (e.g., more specific age categories) [5]. The codebook consisted of categories relevant to equitable inclusion across race, ethnicity, socioeconomic status, pregnancy, disability, and age. Explanations of the potential relevance of these categories to equity, as well as potential scientific and regulatory justifications, are included in Table 1. These categories were selected because they were either directly related to individual demographics (e.g., age, pregnancy) or potentially associated with disproportionate impact on certain groups based on epidemiologic patterns (e.g., HIV disproportionately impacts Black and Hispanic populations) [6]. Several categories were divided into “general” and “specific” subtypes to acknowledge that more specific eligibility criteria may have stronger justifications. Coding was performed by a single author (KS), with audits by other authors (JM, HFL) to ensure consistency and validity; challenging cases were discussed and resolved by all authors.

**Table 1:** Coded eligibility criteria potentially related to equity examined in pivotal trials.

Coded eligibility criterion	Code definition	Example	Relevance to equity	Scientific/Regulatory justification
**Criteria potentially linked to race/ethnicity, socioeconomic, and/or disability status**				
**English language**	Exclusion of individuals unable to speak and/or read English	No example available; not present in dataset	Excluding non-English speakers may lead to greater exclusion of certain racial or ethnic minorities	English language proficiency may be essential to participant compliance with protocol, accurate data collection
**Literacy**	Exclusion of individuals 1) unable to provide and understand written informed consent and/or 2) unable or unwilling to complete patient-reported outcome assessments	*“Ability to provide and understand written informed consent prior to any study procedures” or “Able and willing to undergo frequent MRI or CT assessments and complete symptom assessments using a patient-reported outcome instrument”*	Excluding literacy-limited individuals may lead to greater exclusion of those at lower socio-economic status	Literacy may be essential to participant compliance with protocol, accurate data collection
HIV, General*	Exclusion of individuals with HIV	*“Positive human immunodeficiency virus (HIV) antibody at screening.”*	Excluding individuals with HIV may lead to greater exclusion of certain racial or ethnic minorities, given disproportionate disease burden	HIV may raise concerns about concurrent therapy and/or immune status, especially if not currently controlled
HIV, Specific*	Exclusion of individuals with HIV, with further specification beyond current infection, e.g., time since diagnosis, stable disease, etc.	*“Other ongoing, uncontrolled illnesses (including HIV infection and active hepatitis A, B, or C), psychiatric disorder, or social situation that would prevent good care on this study” [emphasis added]*		
Hepatitis, General*	Exclusion of individuals with hepatitis	*“No Hepatitis C virus (HCV) ongoing infection”*	Excluding individuals with hepatitis may lead to greater exclusion of certain racial or ethnic minorities, given disproportionate disease burden	Hepatitis may raise concerns about concurrent therapy and/or liver function, especially if not currently controlled
Hepatitis, Specific*	Exclusion of individuals with hepatitis, with further specification beyond current infection, e.g., timing, uncontrolled disease, etc.	*“Clinically relevant infection of any kind within the month preceding enrollment (e.g., active hepatitis C, pneumonia)” [emphasis added]*		
Diabetes, General*	Exclusion of individuals with diabetes	*“Have type 1 diabetes mellitus.”*	Excluding individuals with diabetes may lead to greater exclusion of those at lower socioeconomic status and certain racial or ethnic minorities, given disproportionate disease burden	Diabetes may raise concerns about comorbidities and specific physiologic risk factors, especially if not currently controlled
Diabetes, Specific*	Exclusion of individuals with diabetes, with further specification beyond current disease, e.g., type, poorly controlled, etc.	*“Poorly controlled diabetes mellitus” [emphasis added]*		
Hypertension, General*	Exclusion of individuals with hypertension	No example available; not present in dataset	Excluding individuals with hypertension may lead to greater exclusion of those at lower socioeconomic status and certain racial or ethnic minorities, given disproportionate disease burden	Hypertension may raise concerns about comorbidities, concurrent therapy, and specific physiologic risk factors, especially if not currently controlled
Hypertension, Specific*	Exclusion of individuals with hypertension, with further specification beyond current disease, e.g., poorly controlled	*“Uncontrolled blood pressure, defined as systolic blood pressure >180 millimeters of mercury (mmHg) and/or diastolic blood pressure >100 mmHg” [emphasis added]*		
Mental health, General*	Exclusion of individuals with a mental health or psychiatric disorder (other than SUD), without naming specific type	*“Evidence of significant hepatic, renal, respiratory, endocrine, hematologic, neurologic, psychiatric, or cardiovascular (CV) system abnormalities or laboratory abnormality that will affect the health of the subject or interfere with interpretation of the results”*	Excluding individuals with mental health concerns or psychiatric disorders inhibits equitable understanding of drug safety and efficacy to guide clinical care of these populations	Mental health concerns or psychiatric disorders may raise concerns about participant compliance with protocol, comorbidities, concurrent therapy
Mental health, Specific*	Exclusion of individuals with a mental health or psychiatric disorder (other than SUD), with further specification beyond current disease, e.g., type of disorder (by name), acute, unstable, uncontrolled, taking X medications, or a specific time frame	*“Acute or unstable psychiatric conditions diagnosed by the Mini International Neuropsychiatric Interview” [emphasis added]*		
**Weight/obesity**	Exclusion of individuals due to weight or obesity	*“Body mass index below 18.5 or above 40.0 kg/m2”*	Excluding individuals based on obesity may lead to greater exclusion of certain racial or ethnic minorities, given disproportionate disease burden	Obesity may raise concerns about comorbidities, specific physiologic risk factors, changes in pharmacokinetics/dynamics
**Intellectual disability**	Exclusion of individuals with any intellectual disability	*“Mentally incapacitated adults who are incapable of giving legal consent”*	Excluding individuals with intellectual disability inhibits equitable understanding of drug safety and efficacy to guide clinical care of these populations	Intellectual disability may raise concerns about participant compliance with protocol, accurate data collection
Legally authorized representative (LAR)**	Allowance of LAR in informed consent	*“Patients or their legally authorized representative must be willing and able to sign the informed consent form (ICF) and to adhere to the protocol requirements”*	Allowing a LAR to participate in the consent process may allow more equitable participation in research of groups who would otherwise be excluded	Inability to provide direct consent may raise concerns about participant compliance with protocol, accurate data collection
**Smoking**	Exclusion of individuals that smoke, either currently or previously	No example available; not present in dataset	Excluding individuals who smoke may lead to greater exclusion of those at lower socioeconomic status	Smoking may raise concerns about comorbidities, specific physiologic risk factors
**Cannabis use**	Exclusion of individuals that use cannabis currently or previously	*“Participants must have a negative urine drug screen for cannabinoids/ tetrahydrocannabinol and non-prescribed medications at screening.”*	Excluding individuals that use cannabis may lead to greater exclusion of certain racial or ethnic minorities and those at lower socioeconomic status	Cannabis use may raise concerns about specific physiologic risk factors
Substance use disorder (SUD), General*	Exclusion of individuals with SUD	No example available; not present in dataset	Excluding individuals with SUD may lead to greater exclusion of certain racial or ethnic minorities and those at lower socioeconomic status	SUD may raise concerns about participant compliance with protocol, specific physiologic risk factors
SUD, Specific*	Exclusion of individuals with SUD, with further specification beyond current disease, e.g., specific timeline of SUD in relation to study, specific substance named, etc.	*“Current (within 12 months prior to screening) alcohol or drug abuse” [emphasis added]*		
Social condition, General*	Exclusion of individuals based on vague social condition or situation	*“Other ongoing, uncontrolled illnesses (including HIV infection and active hepatitis A, B, or C), psychiatric disorder, or social situation that would prevent good care on this study” [emphasis added]*	Excluding individuals based on their social conditions can facilitate decisions based on implicit bias, which may lead to greater exclusion of certain racial or ethnic minorities and those at lower socioeconomic status; excluding incarcerated individuals raises additional concerns given disproportionate incarceration of certain racial or ethnic minorities	Whether a social condition is scientifically relevant depends on the specific condition; US regulations impose special requirements for research with incarcerated populations
Social condition, Specific*	Exclusion of individuals based on specific social condition or situation	*“Incarcerated individuals”*		
**Sex-related criteria linked to health equity**				
**Pregnancy/Lactation**	Exclusion of individuals who are pregnant, lactating, and/or breastfeeding (not coded if effective birth control required without mention of pregnancy/lactation)	*“Women who are pregnant or breastfeeding”*	Excluding pregnant or lactating individuals inhibits equitable understanding of drug safety and efficacy to guide clinical care of these populations, leading to gender equity concerns	Pregnancy may raise concerns about specific physiologic risk factors, changes in pharmacokinetics/dynamics; Pregnancy/lactation may raise concerns about risk to fetus or child; US regulations impose special requirements for research with pregnant participants
**Criteria linked to health equity across the lifespan**				
**Older adults 65+**	Exclusion of individuals 65 years or older (applied inclusively with 80+ when appropriate)	*“Inclusion Criteria: Between ages of 8-45 years”*	Excluding older individuals inhibits equitable understanding of drug safety and efficacy to guide clinical care of these populations	Older age may raise concerns about comorbidities, specific physiologic risk factors, changes in pharmacokinetics/dynamics
**Older adults 80+**	Exclusion of individuals 80 years or older	*“Inclusion Criteria: Male and female subjects ages 18 to 75 years with…”*		
**Young adults 18–25**	Exclusion of individuals 18–25 years old	*“Ages Eligible for Study: 50 Years and older (Adult, Older Adult)”*	Excluding young adults inhibits equitable understanding of drug safety and efficacy to guide clinical care of these populations	Younger age may influence disease epidemiology and drug pharmacokinetics/dynamics
**Adolescents 12–17**	Exclusion of individuals 12–17 years old (applied inclusively with 18–25 when appropriate)	*“Ages Eligible for Study: 50 Years and older (Adult, Older Adult)”*	Excluding adolescents inhibits equitable understanding of drug safety and efficacy to guide clinical care of these populations	Younger age may influence disease epidemiology and raise concerns about physiologic risk factors, changes in pharmacokinetics/dynamics; US regulations impose special requirements for pediatric research

Several categories were divided into “general” and “specific” subtypes to acknowledge that more specific eligibility criteria may have stronger justifications. All eligibility criteria represented in this table exclude individuals from participating, except for LAR, which renders individuals eligible to participate.

We calculated the frequency of equity-related eligibility criteria overall and across several drug categories (orphan; oncology; first-in-class; expedited). We included expedited programs (breakthrough, fast track, and accelerated approval) as FDA has deemed these drugs promising and likely to address unmet treatment needs, increasing the importance of equitable opportunities for trial participation and appropriately generalizable study populations. Due to the small number of trials with relevant exclusion criteria in these categories, we did not assess for statistically significant differences between them.

## Results

In 2022, CDER approved 37 novel drugs based on a total of 56 pivotal trials. As described in Table 2, the most common equity-related exclusion criteria were of adolescents (45, 80.4%), pregnant/lactating individuals (30, 53.6%), and individuals with limited literacy (20, 35.7%); this pattern held across drug categories. About 1 trial in 5 excluded individuals based on weight/obesity (11, 19.6%), had general exclusions for hepatitis, i.e., without specification beyond current infection (10, 17.9%), or excluded adults aged 80 and up (11, 19.6%). General HIV exclusions were present in 7 (12.5%) trials. With few exceptions, exclusions based on literacy, HIV, and hepatitis were proportionately more common for trials supporting specific drug categories than they were in pivotal trials overall. Pregnant/lactating individuals were most often excluded from trials supporting drugs with fast-track designation (10, 71.4%) and those granted accelerated approval (4, 66.7%).

**Table 2:** Frequency of equity-related eligibility criteria examined in pivotal trials, overall and by drug/approval type.

		Overall Trial Frequency (n = 37 drugs; n = 56 trials)	Drugs with Orphan Designation (n = 20 drugs; n = 20 trials)	Oncology Drugs (n = 11 drugs; n = 12 trials)	First-In-Class Drugs (n = 20 drugs; n = 27 trials)	Drugs with Breakthrough Designation (n = 13 drugs; n = 14 trials)	Drugs with Fast Track Designation (n = 12 drugs; n = 14 trials)	Drugs Granted Accelerated Approval (n = 6 drugs; n = 6 trials)
**Theme**	**Coded eligibility criteria, n, trials (%)**							
	**Criteria potentially linked to race/ethnicity, socioeconomic, and/or disability status**							
**Language and Literacy related exclusions**	**English language**	0	0	0	0	0	0	0
	**Literacy**	20 (35.7)	10 (50.0)	5 (41.7)	12 (44.4)	6 (42.9)	6 (42.9)	4 (66.7)
**Co-Morbidity based exclusions**	**HIV, General**	7 (12.5)	5 (25.0)	2 (16.7)	6 (22.2)	4 (28.6)	4 (28.6)	2 (33.3)
	**HIV, Specific**	2 (3.6)	2 (10.0)	1 (8.3)	1 (3.7)	1 (7.1)	1 (7.1)	1 (16.7)
	**Hepatitis, General**	10 (17.9)	5 (25.0)	3 (25.0)	8 (29.6)	4 (28.6)	5 (35.7)	2 (33.3)
	**Hepatitis, Specific**	8 (14.3)	4 (20.0)	2 (16.7)	7 (25.9)	4 (28.6)	1 (7.1)	1 (16.7)
	**Diabetes, General**	5 (8.9)	0	0	5 (18.5)	0	0	0
	**Diabetes, Specific**	4 (7.1)	1 (5.0)	0	0	0	3 (21.4)	0
	**Hypertension, General**	0	0	0	0	0	0	0
	**Hypertension, Specific**	4 (7.1)	1 (5.0)	0	0	0	0	0
	**Mental health, General**	2 (3.6)	0	0	2 (7.4)	0	0	0
	**Mental health, Specific**	5 (8.9)	3 (15.0)	0	0	0	1 (7.1)	1 (16.7)
	**Weight/obesity**	11 (19.6)	3 (15.0)	0	7 (25.9)	3 (21.4)	0	0
**Intellectual disability**	**Intellectual disability**	2 (3.6)	2 (10.0)	0	0	0	0	0
	**LAR (inclusion)**	1 (1.8)	1 (5.0)	1 (8.3)	1 (3.7)	0	1 (7.1)	1 (16.7)
**Substance use related exclusion**	**Smoking**	0	0	0	0	0	0	0
	**Cannabis use**	1 (1.8)	0	0	1 (3.9)	0	1 (7.1)	0
	**SUD, General**	0	0	0	0	0	0	0
	**SUD, Specific**	3 (5.4)	2 (10.0)	0	2 (7.4)	1 (7.1)	2 (14.3)	0
**Social condition related exclusion**	**Social condition, General**	4 (7.1)	1 (5.0)	0	0	0	1 (7.1)	1 (16.7)
	**Social condition, Specific**	2 (3.6)	2 (10.0)	1 (8.3)	1 (3.7)	1 (7.1)	0	0
	**Sex-related criteria linked to health equity**							
	**Pregnancy/ lactation**	30 (53.6)	10 (50.0)	4 (33.3)	12 (44.4)	7 (50.0)	10 (71.4)	4 (66.7)
	**Criteria linked to health equity across the lifespan**							
**Age related exclusions**	**Older adults 65+**	4 (7.1)	1 (5.0)	0	2 (7.4)	1 (7.1)	0	0
	**Older adults 80+**	11 (20.0)	4 (20.0)	0	5 (18.5)	2 (14.3)	0	0
	**Young adults 18–25**	3 (5.4)	1 (5.0)	0	1 (3.7)	0	0	0
	**Adolescents 12–17**	45 (80.4)	18 (90.0)	11 (91.7)	23 (85.2)	10 (71.4)	9 (64.3)	6 (100.0)

HIV = human immunodeficiency virus; LAR = legally authorized representative; SUD = substance use disorder.

Criteria allowing exclusion based on an individual’s general social condition were infrequent (4, 7.1%), as were those excluding adults aged 65 and up (4, 7.1%), young adults (3, 5.4%), or individuals with a general mental health condition (2, 3.6%). Although individuals with intellectual disability were infrequently excluded (2, 3.6%), only 1 trial explicitly permitted a Legally Authorized Representative to consent. No trials excluded individuals based on general substance use disorder or smoking; only 1 excluded for cannabis use. While no trial explicitly required English language, this likely overlaps with common literacy exclusions.

## Discussion

Our analysis found that eligibility criteria in pivotal trials supporting 2022 drug approvals were broadly inclusive of individuals with HIV, diabetes, hypertension, mental health conditions, and substance use disorder, as well as those who smoke, use cannabis or are intellectually disabled. Most trials avoided vague “catch-all” exclusions based on general social condition that could lead to bias based on factors like race, ethnicity, or socioeconomic status. For oncology studies and accelerated approvals (which often overlap), several trials had no equity-related exclusion criteria beyond literacy, pregnancy/lactation, and age.

Exclusion of adolescents was common but likely reflects distinct safety considerations and regulations applicable to pediatric research, as well as distinct disease epidemiology; in addition, children may be more often included in post-approval studies that are encouraged or required under FDA policy. Exclusion based on pregnancy/lactation status was also common, likely for similar reasons, despite expert recommendations encouraging routine inclusion of pregnant/lactating people in later stage studies, such as pivotal trials supporting drug approval [7,8]. Frequent exclusion based on weight/obesity documented in our sample could have clinical justification, for example related to dosing or various risk factors, but more specific rationales for exclusion would be most appropriate. While current diversity discussions have largely focused on race/ethnicity, sex, and age, there has been less attention to exclusion based on body size despite high rates of overweight and obesity in the American population. Similarly, the substantial exclusion of adults aged 80 and up found in our sample sometimes may be clinically justified but warrants attention given the aging population and need for data to support use in Medicare beneficiaries. Finally, the frequent exclusion of individuals with limited literacy reflected in our findings could be addressed by utilizing recommended literacy supports such as ensuring that all research materials, including consent forms, are written in plain language, utilizing visual aids like diagrams and videos, facilitating reading/writing assistance, and offering individualized support from research team members. Similar strategies can be employed for individuals with cognitive impairment, as well as permitting reliance on legally authorized representatives (LARs) [9].

### Limitations

Our findings are limited by inclusion of only 1 year of FDA drug approvals, precluding comparison across drug categories and identification of trends over time. However, this approach offered a snapshot of trials supporting FDA approvals immediately before a moment of substantial policymaking intended to encourage trial diversity. Evaluating whether eligibility criteria are justified is complex and depends on nuanced scientific and regulatory considerations. Because we were unable to evaluate whether eligibility criteria were justified in the context of each trial, our findings may overestimate equity-related exclusions.

## Conclusion

FDA guidance recommends broadening eligibility criteria when scientifically appropriate to improve diverse participation, recognizing that some criteria have become “commonly accepted over time or used as a template across trials… excluding certain populations... without strong clinical or scientific justification” [10]. While overt exclusion based on race/ethnicity and socioeconomic status is not reflected in eligibility criteria, it is important to also consider associated factors that could lead to the same outcome, such as exclusion based on comorbidities that reflect health disparities. It is also essential to address trial diversity across all domains that influence the generalizability of study findings and relate to fair distribution of benefit. To minimize unnecessary equity-related exclusions, eligibility should focus on specific issues expected to influence safety or efficacy (e.g., liver function, drug interaction, uncontrolled comorbid disease), rather than blanket exclusion based on broad diagnoses or characteristics. Accommodations also may be possible. Investigators, sponsors, institutional review boards, and regulators should scrutinize eligibility criteria with equity in mind.
